# Supplementary material for: A Rapid-prototyping CMOS-RRAM Integration Strategy
Source: Microsyst Nanoeng. 2026 May 26;12:206. doi: 10.1038/s41378-026-01335-9 (PMC13212575; doi:10.1038/s41378-026-01335-9)
Supplement: Supplementary file 1 — Supplementary Material - A Rapid-prototyping CMOS-RRAM Integration Strategy [file 41378_2026_1335_MOESM1_ESM.docx]

**Supplementary Information**

**A Rapid-prototyping CMOS-RRAM Integration Strategy**

Andreas Tsiamis^1*^, Spyros Stathopoulos^1^ and Themis Prodromakis^1^

^1^ Centre for Electronics Frontiers, Institute for Integrated Micro and Nano Systems, School of Engineering, The University of Edinburgh, Edinburgh, EH9 3BF, U.K. (email: a.tsiamis@ed.ac.uk, s.stathopoulos@ed.ac.uk and t.prodromakis@ed.ac.uk)

^*^ Corresponding author (Andreas Tsiamis).

Address: Scottish Microelectronics Centre, Alexander Crum Brown Road, King's Buildings, Edinburgh, UK. EH9 3FF

Telephone: +44 131 6505632

**Automated pipeline for detecting etched features in optical images**

We have developed an automated pipeline for detecting etched features in optical microscopy images, specifically targeting circular dots (holes), in particular passivation openings that in this case allow to create RRAM connectivity openings to the top CMOS metallisation. The detection method verifies the success of this integration process step and allows to quantify yield.

The method employs a two-stage filtering process to robustly isolate etchings from background noise and artefacts. In the first stage, a brightness-based filter is applied. Etched regions typically appear as the brightest areas in the image; we retain only the top percentile of pixel intensities, defined by a configurable parameter p_b, effectively suppressing background regions. The brightness threshold T_b is determined from the cumulative distribution function (CDF) of the grayscale image histogram as:

T_b = min { t | CDF(t) >= p_b }

Pixels above this threshold are segmented as candidate etch regions.

The second stage leverages the circularity of the etched features. Each candidate contour is quantified by its circularity metric C, defined as:

C = (4 × π × A) / (P × P)

where A is the area and P the perimeter of the contour. Perfect circles have C = 1, whereas elongated or irregular shapes yield lower values. Contours exceeding a configurable circularity threshold C_t are considered valid etchings. This step removes spurious noise that passes the brightness filter but does not match the expected circular morphology.

The pipeline produces a set of annotated outputs for each image, including detected etch locations overlaid on the original image, grayscale and binary intermediate representations, and histograms of both brightness and circularity. A composite analysis report visualizes all processing stages, enabling quantitative assessment of detection accuracy and providing a reproducible method for automated etch inspection in optical imaging.

**
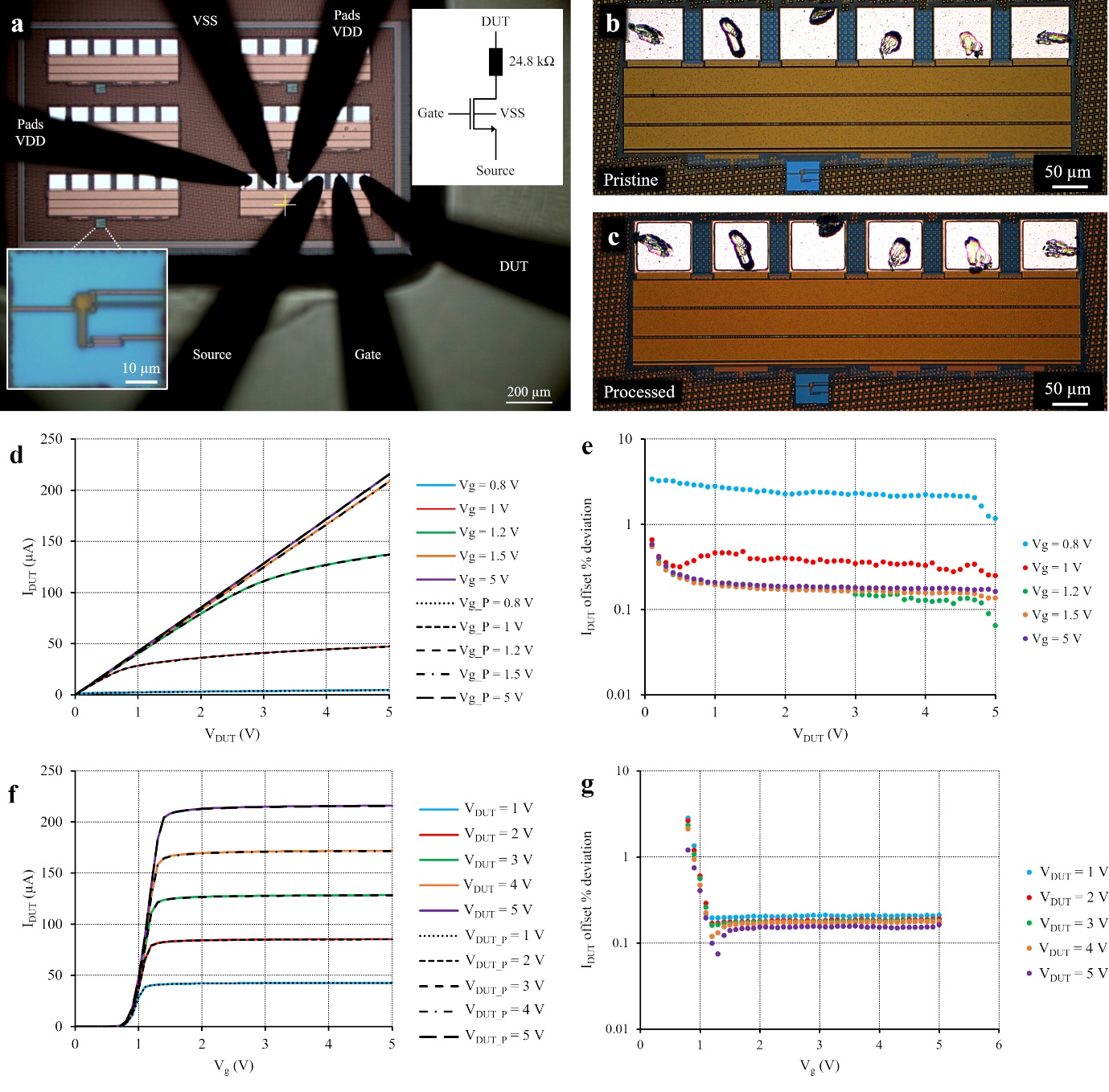
**

**Fig. S1: Electrical characterisation and optical validation of an on-reticle nMOS cell before and after post-processing.** (a) Measurement setup (optical imaging) and schematic diagram of the nMOS cell. (b) Optical imaging of the pristine device. (c) Optical imaging of the post-processed device. (d) I_DUT_ vs V_DUT_ (device under test) for a number of gate voltages V_g_ (pristine) / V_g_P_ (post-processed) for both the pristine (solid lines) and the post-processed device (dashed lines). (e) I_DUT_ offset between the two measurements presented as a percentage deviation from the pristine I_DUT_ measurement vs V_DUT_ for a number of Vg. (f) I_DUT_ vs V_g_ for a number of V_DUT_ (pristine) / V_DUT_P_ (post-processed) for both the pristine (solid lines) and post-processed device (dashed lines). (g) I_DUT_ offset between the two measurements presented as a percentage deviation from the pristine I_DUT_ measurement vs V_g_ for a number of V_DUT_


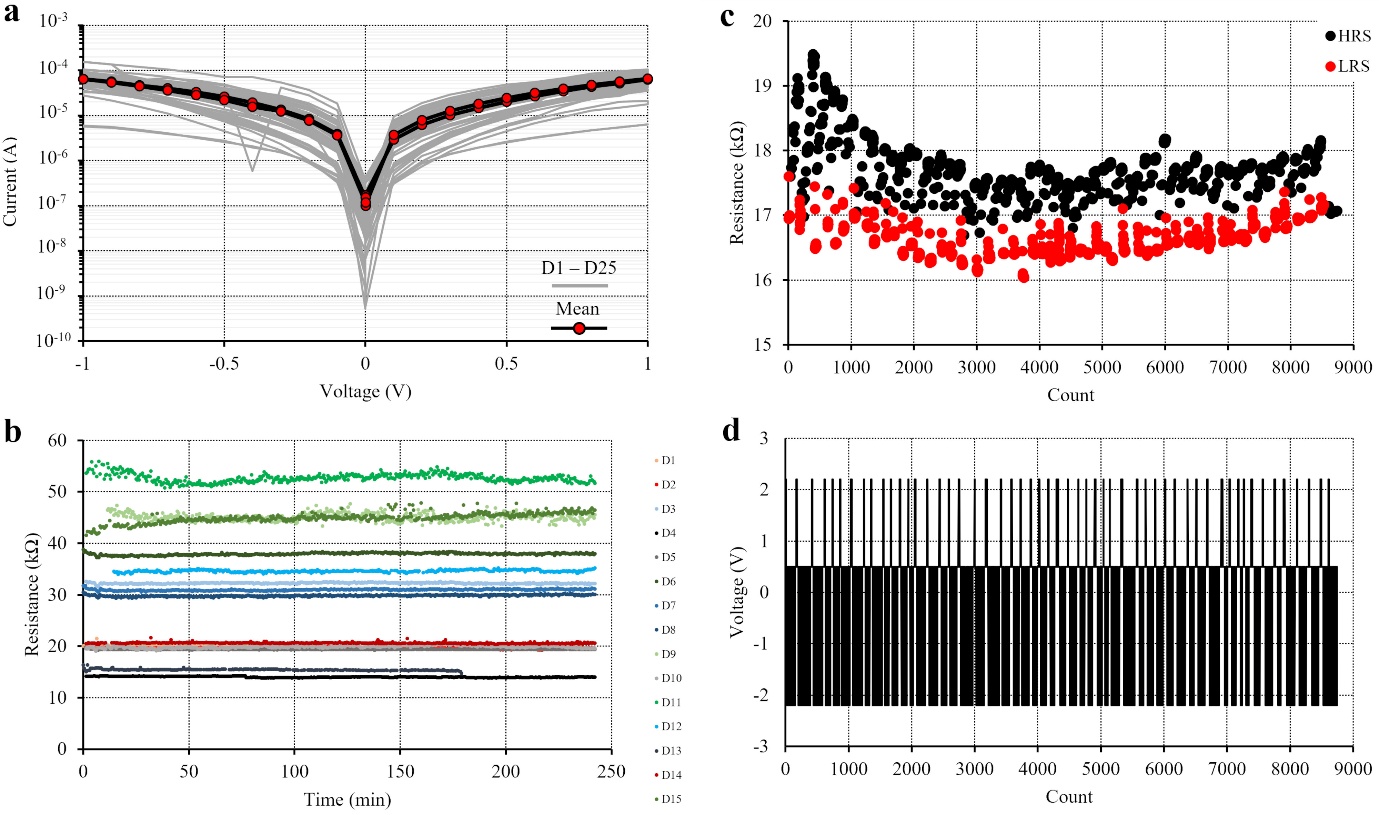


**Fig. S2: Electrical characterisation of CMOS integrated RRAM.** (a) I–V characteristic curves (1_st_ cycle) of TiN/HfO_x_N_y_/TiN RRAM illustrating device-to-device variability (n_device_ = 25). (b) Retention measurements of distinct devices programmed at several resistance states (n_device_ = 15, V_read_ = 0.5 V, retention time = 240 min). (c) Prolonged analogue RRAM cycling. (d) RRAM cycling pulsing sequence (V_pulse_ = ± 2.2 V, V_read_ = 0.5 V)

**
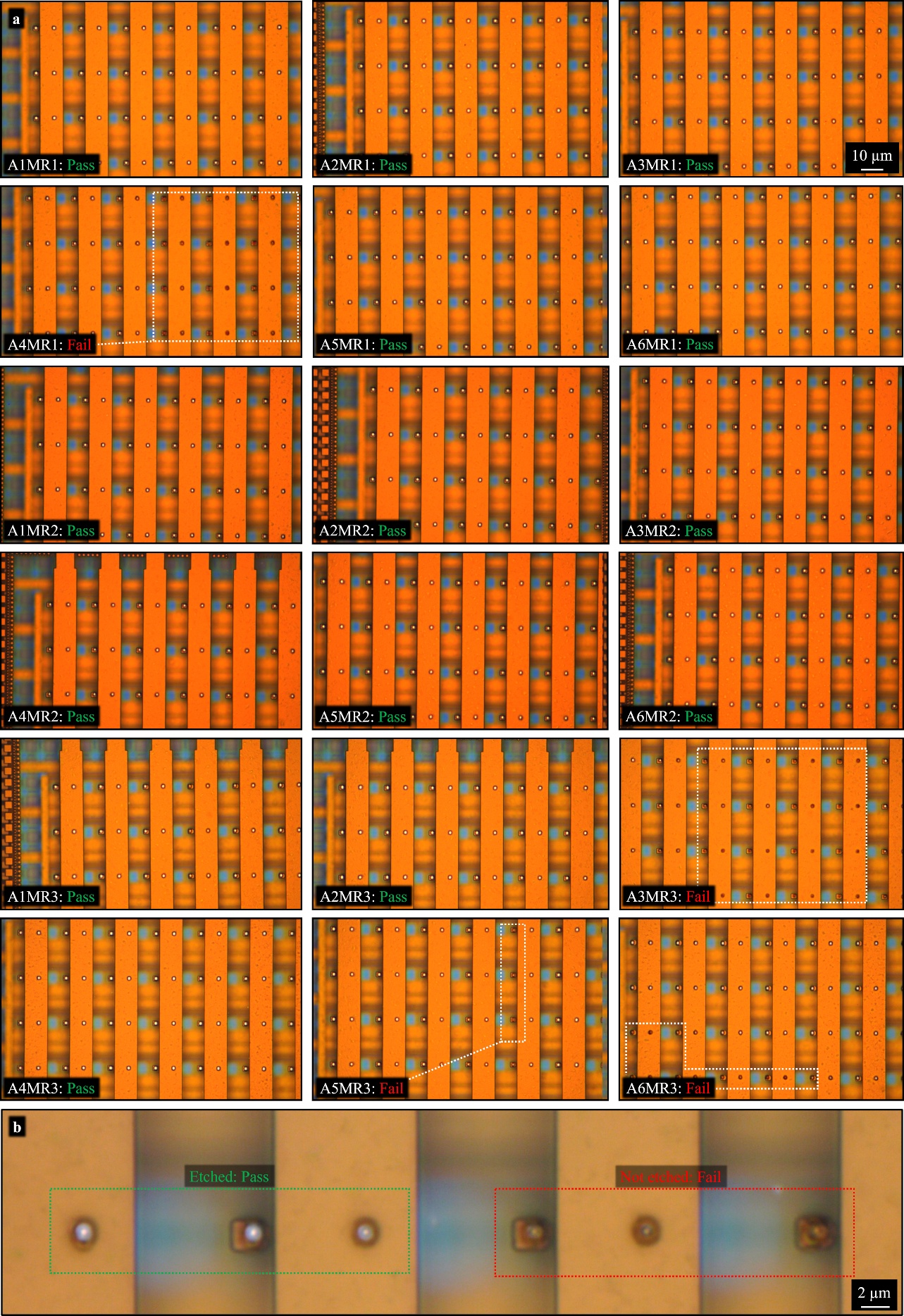
**

**Fig. S3: RRAM integration yield analysis at the passivation etch stage.** (a) Optical imaging of 18 arrays that have been etched to create RRAM connectivity openings. Arrays that have all the openings fully etched, pass (~78% yield), while arrays that have any number of openings partially etched, fail. (b) Optical image illustrating passivation openings that have been fully etched and pass the yield test (left) and openings that have been partially etched and fail the yield test (right). A bright white opening indicates that the underlying CMOS metallisation has been exposed, while a darker opening indicates a partially etched feature

**
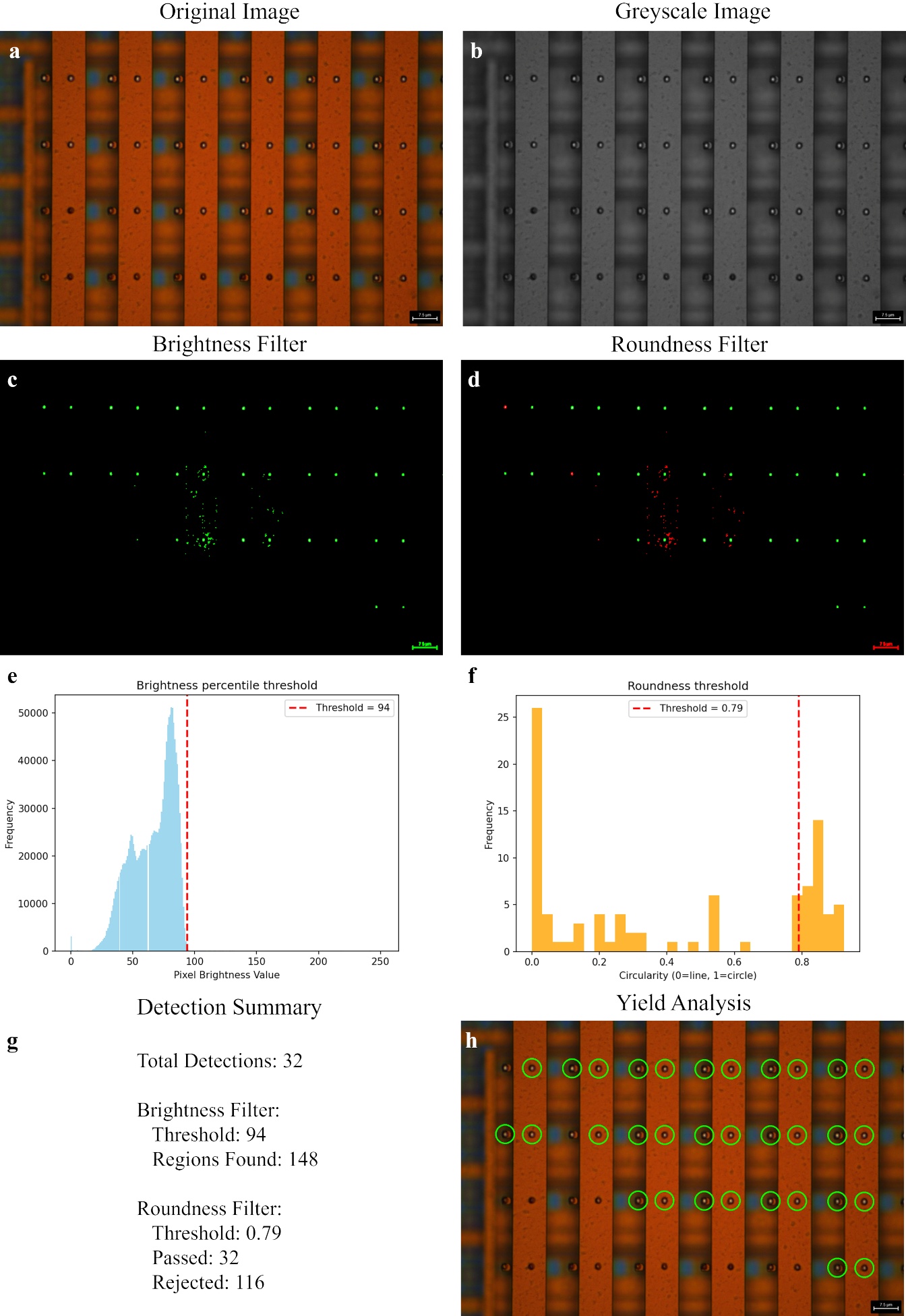
**

**Fig. S4: Automated detection of etched features in optical images.** Demonstration with array A6MR3. (a) Original image. (b) Greyscale image. (c) Brightness filter. (d) Roundness filter. (e) Brightness percentile threshold. (f) Roundness threshold. (g) Detection summary. (h) Yield analysis (green highlights fully etched openings)


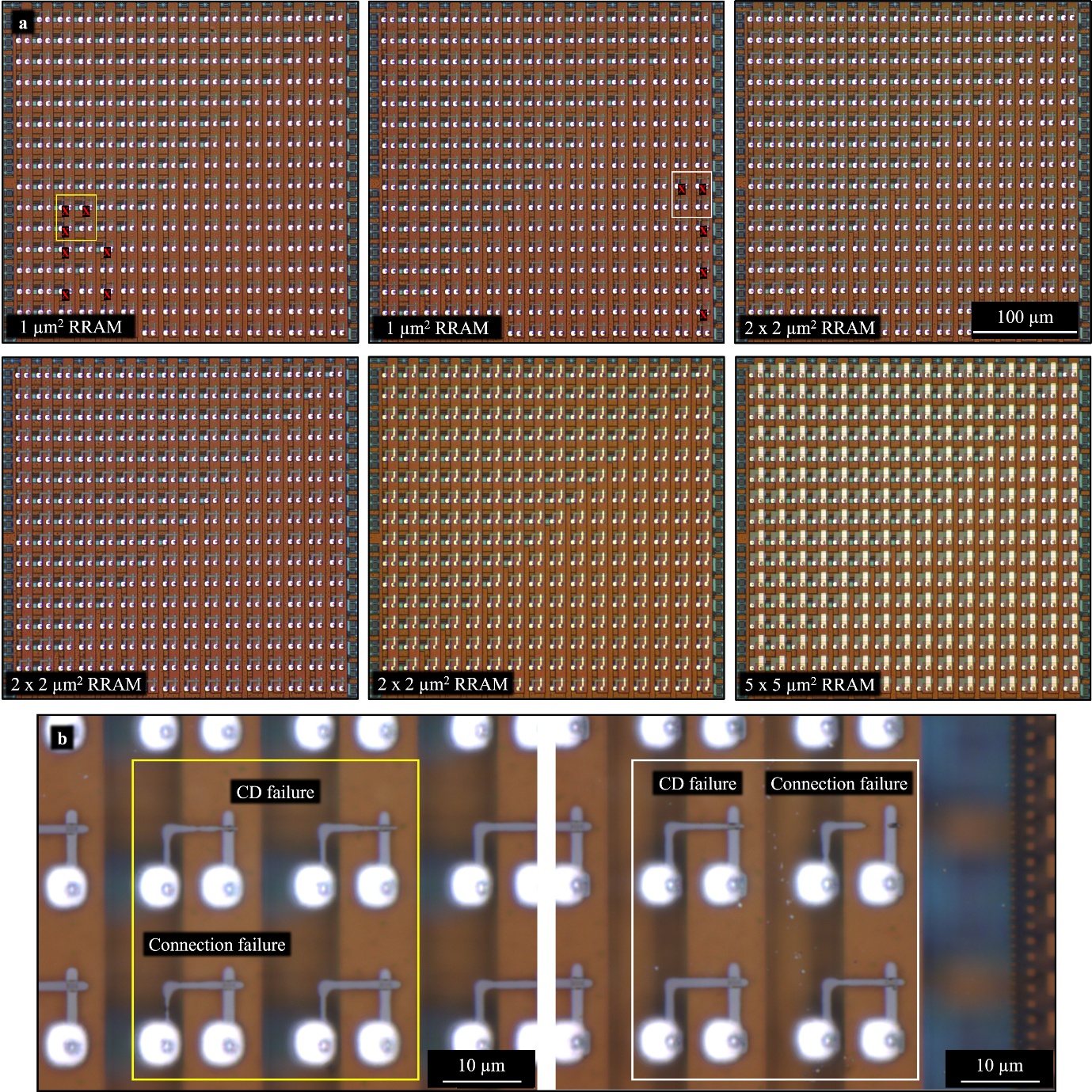


**Fig. S5: RRAM integration yield analysis at the full-device stage.** (a) Optical imaging of six RRAM arrays with dimensions ranging from 1 to 5 x 5 µm^2^. Arrays with larger RRAM dimensions are defect-free, while a ~98% fabrication yield is observed for the smallest sized arrays. (b) Common RRAM failures appear as loss of connectivity between RRAM and CMOS or as a significant mismatch between nominal and fabricated device (active area) dimension (these devices are also highlighted as rectangular sections on the images of the full arrays)

**
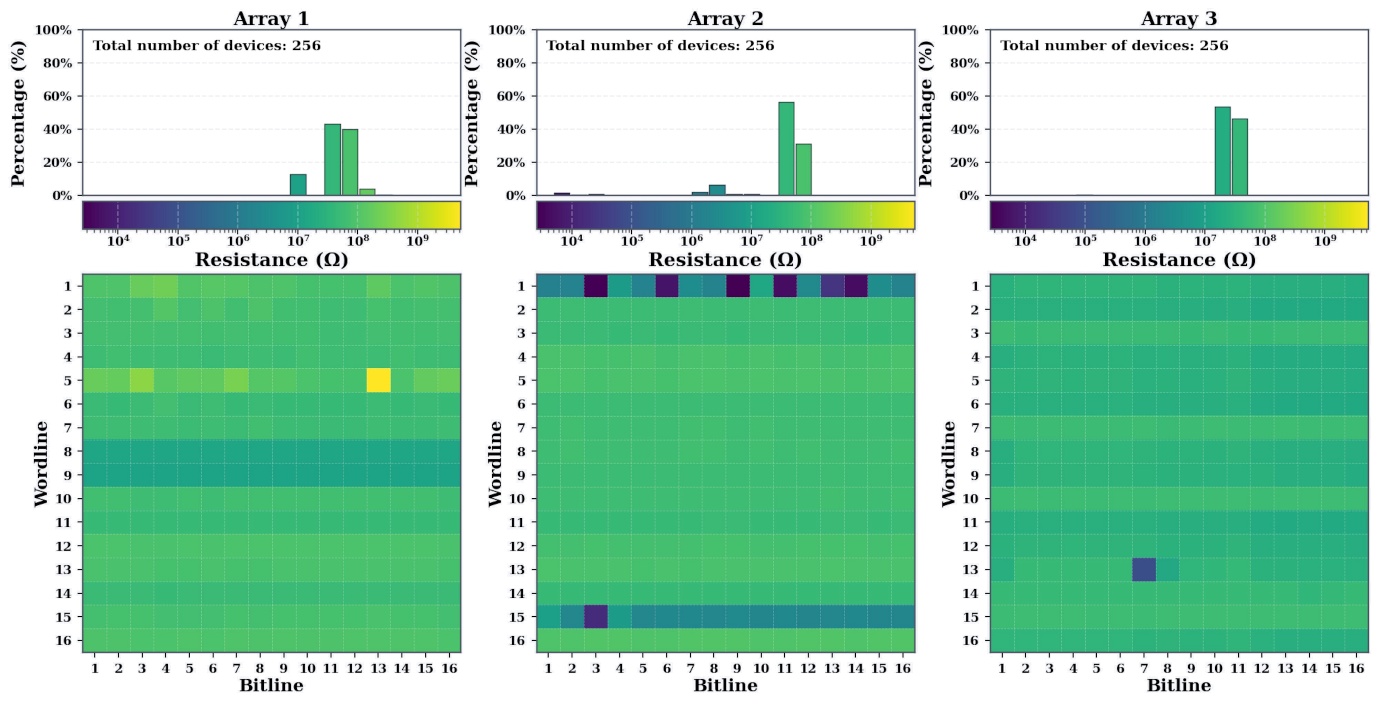
**

**Fig. S6: Electrical yield analysis of process-inspected defect-free 2 x 2 µm^2^ CMOS-RRAM integrated arrays with devices at their pristine state.** Statistical distribution of measured resistance (V_read_ = 0.5 V) for arrays 1-3 (top). Resistance maps (V_read_ = 0.5 V) for arrays 1-3 (bottom)


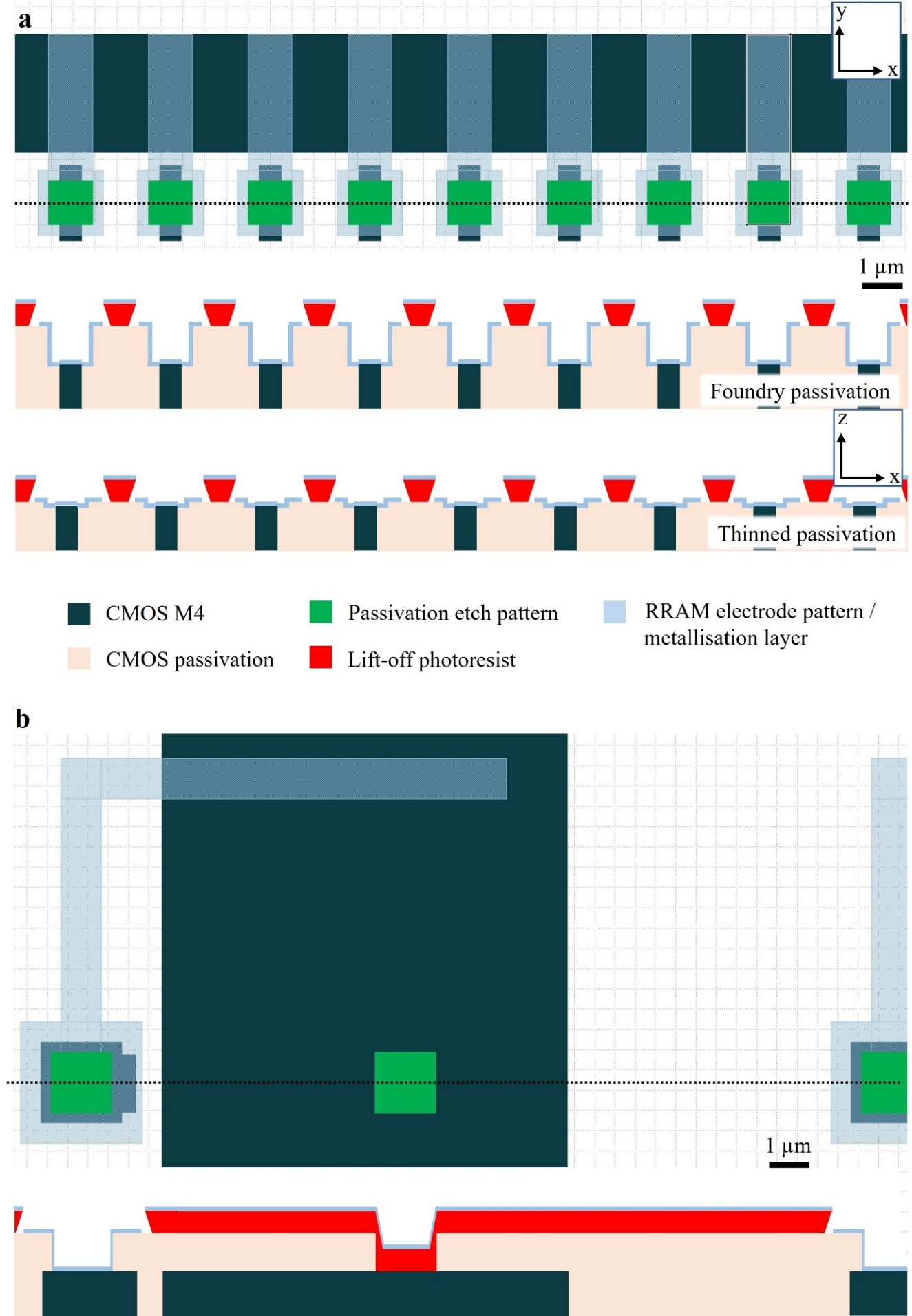


**Fig. S7: Photolithographic patterning of high- and low-density arrays on deep and shallow surface topologies.** (a) Layout snippet of dense array (515 x 512 1T1R) patterning (top) and cross-sectional schematics of desired post-metallisation patterning on deep (middle) and shallow (bottom) trench topologies. (b) Layout snippet of low-density (16 x 16 1T1R) array patterning (top) and cross-sectional schematic of desired post-metallisation patterning on deep (bottom) trench topology


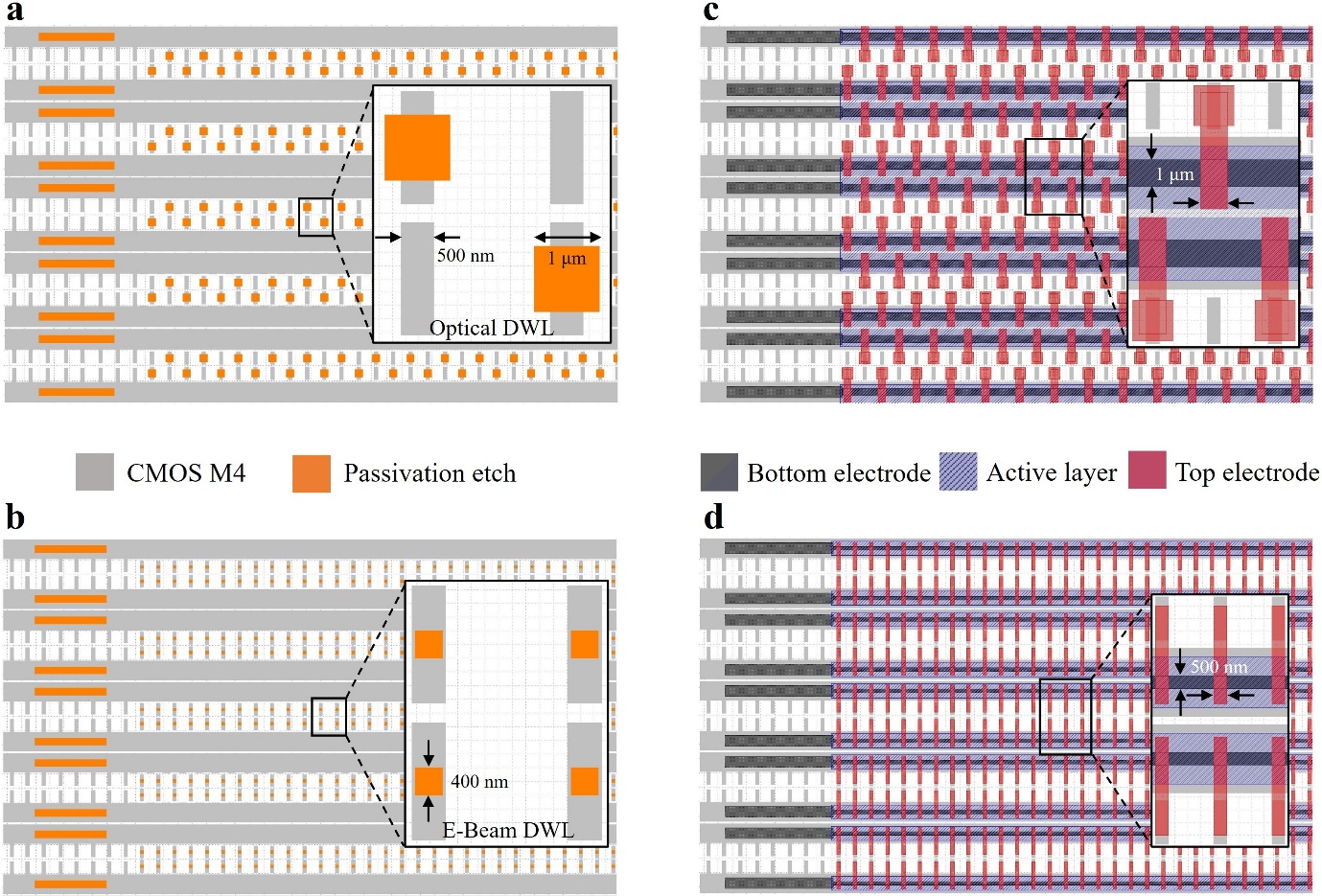


**Fig. S8: 1Mbit 1T1R integration layout (virtual masks), designed for optical and e-beam DWL.** Lithographic patterns for a (a) half-array passivation etch (optical), (b) full-array passivation etch (e-beam), (c) half-array RRAM stack (optical) and (d) full-array RRAM stack (e-beam)


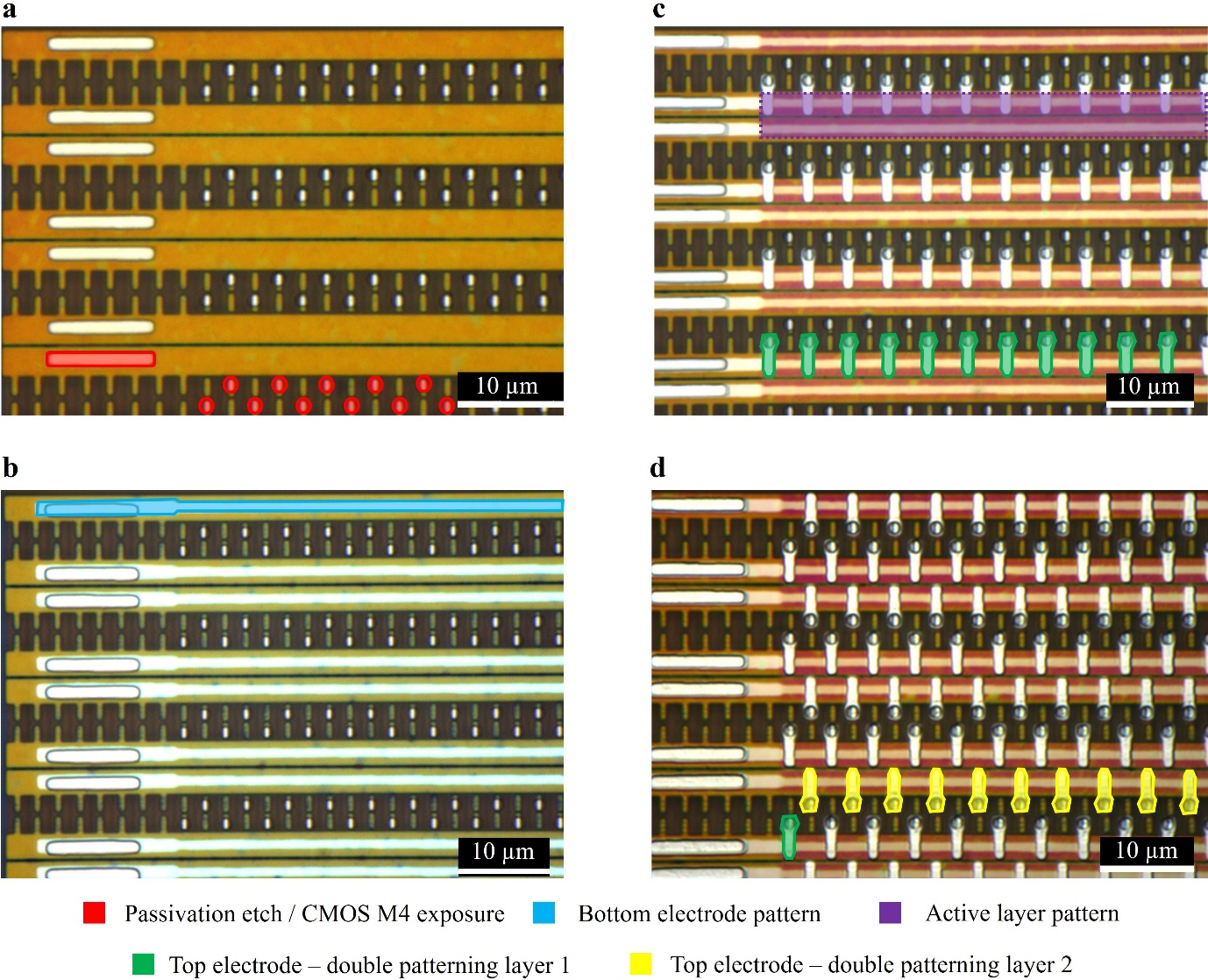


**Fig. S9: 0.5Mbit 1T1R integration using optical DWL.** (a) Foundry passivation is etched to expose the top CMOS metal. Large openings allow to connect to bottom RRAM electrodes, while small openings allow to connect to top electrodes. (b) Common (per row) bottom electrode patterning. (c) RRAM active layer patterning and patterning of half of the top RRAM electrode features (layer 1). (d) Double patterning employed (layer 2) to fabricate the remaining half of the top RRAM electrode features
